# HIV fatalism and engagement in transactional sex among Ugandan fisherfolk living with HIV

**DOI:** 10.1080/17290376.2019.1572533

**Published:** 2019-02-06

**Authors:** Katelyn M. Sileo, Laura M. Bogart, Glenn J. Wagner, William Musoke, Rose Naigino, Barbara Mukasa, Rhoda K. Wanyenze

**Affiliations:** a Division of Global Health, University of California San Diego, La Jolla, CA, USA; b RAND Corporation, Santa Monica, CA, USA; c Division of General Pediatrics, Department of Medicine, Boston Children’s Hospital, Boston, MA, USA; d Department of Pediatrics, Harvard Medical School, Boston, MA, USA; e Mildmay Uganda, Kampala, Uganda; f Department of Disease Control and Environmental Health, Makerere University, Kampala, Uganda

**Keywords:** HIV, fatalism, transactional sex, sexual risk, fisherfolk, Uganda

## Abstract

HIV fatalism, or the belief that HIV acquisition and mortality is out of one's control, is thought to contribute to HIV risk in fishing populations in East Africa. The objective of this cross-sectional study was to investigate the association between fatalism and sexual risk behaviours (unprotected sex, engagement in transactional sex), beyond the influence of other known HIV risk factors (e.g. food insecurity, mobility), and identify demographic, psychosocial, and structural correlates of HIV fatalism. Ninety-one men and women living in fishing villages on two islands in Lake Victoria, Uganda completed an interviewer-administered questionnaire after testing HIV-positive during home or community-based HIV testing between May and July 2015. Multivariate logistic regression was used to test the association between HIV fatalism and transactional sex and multivariate linear regression was used to identify demographic, psychosocial, and structural correlates of HIV fatalism. HIV fatalism was significantly associated with a greater likelihood of transactional sex (AOR = 3.07, 95% CI = 1.02–9.23, *p* = 0.04), and structural barriers to HIV care (e.g. distance to clinic) were significantly associated with HIV fatalism (*β* = 0.26, *SE* = 0.12, *p* = 0.04). Our findings highlight HIV fatalism as a contributor to transactional sex in Ugandan fishing communities, and as a product of broader social and contextual factors, suggesting the potential need for structural HIV interventions in this setting.

## Introduction

Fisherfolk are among the most at-risk groups for HIV in the world (Kissling et al., 2005). Fisherfolk include fishermen, fish traders and sellers, and other individuals supporting the fishing industry. In a multi-country analysis comparing fisherfolk to other high risk sub-populations such as truck drivers, injection drug users, and sex workers, HIV prevalence was similar or higher among fisherfolk (Kissling et al., 2005). For example, HIV prevalence estimates from East African countries were between 20.30–30.50% for fisherfolk, compared to 13.50–14.80% for truck drivers and 27.60–47.00% for sex workers from the region (Kissling et al., 2005). In the fishing communities surrounding Lake Victoria, Uganda, HIV prevalence is estimated to be high as 35.00% (Asiki et al., 2011; Opio, Muyonga, & Mulumba, 2011; Seeley et al., 2012) compared to 6.5% in the general adult population (UNAIDS, 2018), and high rates of risky sexual behaviour are reported (Asiki et al., 2011; Kiwanuka et al., 2013; Seeley et al., 2012). Commercial sex work and engagement in transactional sex (sex in exchange for money or goods) in particular have been identified as major contributors to the HIV epidemic in these communities (MacPherson et al., 2012; Sileo, Kintu, Chanes-Mora, & Kiene, 2016; Smolak, 2014). In a meta-analysis of 44 studies with fisherfolk in Uganda and similar developing countries, 42% of fishermen had engaged in transactional sex, 48% of whom reported unprotected sex with female sex workers (Smolak, 2014).

HIV fatalism is thought to contribute to HIV risk behaviour in fishing populations (Hess & McKinney, 2007; Meyer-Weitz, 2005). HIV fatalism is defined as the belief that HIV acquisition and mortality is out of one's control, and is consistent with the Powe Fatalism Model which posits that people are more likely to engage in health promotion when they believe they are in control of their own health (Powe, 1995, 1997). HIV fatalism among fishermen is believed to develop in response to exposure to occupational hazards. In Uganda and similar settings, fishing is a dangerous occupation and in qualitative research, fishermen cite the fatalistic belief that they are more likely to die fishing than of HIV (Lubega et al., 2015; Mojola, 2011; Sileo et al., 2016). These occupational risks appear to distort fishermen's perception of their risk of HIV-related death, potentially leading to apathy towards acquiring HIV or the denial of HIV risk (Kissling et al., 2005; Lubega et al., 2015; Mojola, 2011). HIV fatalism may therefore limit motivation to engage in HIV prevention, testing, or treatment. Fatalism may also lead to increased engagement in transactional sex or sex with multiple partners as a way to cope with the daily stresses of working in a dangerous occupation (Sileo et al., 2016).

HIV fatalism appears to emerge in communities with a high incidence of AIDS-related deaths and inadequate support for HIV prevention, as well as in ‘circumstances of social alienation and disintegration’ (Meyer-Weitz, 2005). Fishermen, fish traders, and sex workers are often highly marginalised, frequently moving around the lake for work and separated from their families (Allison & Seeley, 2004; Seeley, Tumwekwase, & Grosskurth, 2009). This isolation, paired with the high burden of HIV, may make fatalism especially prevalent among Ugandan fishing communities, even for those not directly involved in fishing.

As described in the above-cited qualitative literature, HIV fatalism is reportedly prevalent among Ugandan fisherfolk, and may contribute to HIV risk behaviour through the perception that HIV is inevitable or by distorting fishermen's risk perceptions of HIV-related death compared to other occupational hazards (Lubega et al., 2015; Seeley et al., 2009; Sileo et al., 2016). However, to our knowledge, no studies have quantitatively examined the level and types of fatalistic beliefs among Ugandan fisherfolk, or the association between fatalism and sexual risk. Moreover, more research is needed to assess factors associated with HIV fatalism in this context to inform the targeted and tailored development of HIV risk reduction interventions for fisherfolk. The overall objective of this paper is to investigate the association between fatalism and sexual risk behaviours (unprotected sex, engagement in transactional sex), beyond the influence of other known HIV risk factors. We aimed to test socio-demographic (sex, age, education, monthly household expenditure, occupation), psychosocial (internalised and anticipated HIV stigma), and structural (food insecurity, mobility) HIV risk factors (Kaufman, Cornish, Zimmerman, & Johnson, 2014) as independent correlates of sexual risk, and adjust for these factors in multivariate models assessing the effect of fatalism on sexual risk behaviours. We further aimed to identify demographic, psychosocial, and structural correlates of HIV fatalism. Given the above-cited qualitative literature, we hypothesised the same psychosocial and structural factors may be related to HIV fatalism, with the additions of perceived poor health (psychosocial) and structural barriers to HIV care (structural).

## Methods

### Participants and setting

Data were from a larger study evaluating uptake of HIV testing and linkage to HIV care among fisherfolk offered two types of community-based HIV testing in partnership with Mildmay Uganda, a nonprofit organisation providing free HIV testing and treatment in Uganda: home-based HIV counseling and testing (HBHCT) and outreach event-based HIV counseling and testing (Bogart et al., 2017). HBHCT consists of medical staff and HIV counselors going door-to-door throughout communities to offer HIV testing and counseling in the home. Outreach event-based testing includes clinic staff conducting outreach events outside of clinic structures in communities, to educate people about HIV testing, and to provide HIV counseling and testing services.

The study took place in two Ugandan fishing communities in Wakiso District, Lake Victoria: Kavenyanja Island (in Kachanga Village), where residents were offered home-based testing, and Zzinga Island, where individuals volunteered to be tested during outreach events. As per our protocol, HIV testing counsellors did not refer participants to interviewers unless participants agreed to be interviewed and referred; all clients who tested positive during home or event-based testing agreed to be referred and were therefore offered participation. To protect confidentiality of participants who did not want to enroll in the study, the interviewers were not present when HIV counsellors revealed the results. Participants had a choice of whether to do the interview immediately after testing or to schedule the interview for a later time, which allowed participants time to emotionally process their result if needed before the interview. The ethical protocol and counsellor training specified that participants who appeared to be very distressed should not be referred for the interview.

On Kavenyanja Island, 1,364 residents were offered and 822 received HBHCT, and 82 clients screened positive for the first time during HBHCT; of these 82 clients, 60 completed a survey. Of 344 fisherfolk tested during one of eight event-based testing efforts on Zzinga Island, 33 were newly diagnosed positive, of whom 32 were eligible for the interview (i.e. over 18 years of age) and 31 completed the survey (1 missed the appointment). Therefore, a total of 91 participants (51 female, 40 male) completed the survey and are included in the present analysis.

### Procedures

In Kachanga, Kavenyanja, all household residents were offered HBHCT 4 days per week for 8 weeks (May-July 2015). Interviewers accompanied HIV counselors to households to recruit individuals who screened positive for participation in the survey. HIV counselors conducted HIV testing and counseling following the standard Ministry of Health (MOH) procedures for HBHCT (Uganda MOH, 2005). For event-based testing on Zzinga Island, rapid HIV testing was provided during eight community outreach mobilisation events during May-July, 2015. In both testing settings, all individuals 18 years of age or older who screened newly positive for HIV were offered participation in a questionnaire immediately after receipt of HIV test results. Interviewers administered questionnaires programmed on tablets using Questionnaire Design Studio 3.0 in a private setting (a church or homes in Kachanga; clinic or homes on Zzinga) immediately after HIV test results were delivered in both testing sites. Participants were compensated 10,000 Ugandan Shillings (∼ 3 USD) for participating in the survey. All participants provided written informed consent following procedures approved by the Institutional Review Boards at Boston Children's Hospital and Makerere University School of Public Health. The study was also approved by the Uganda National Council for Science and Technology.

### Measures

*Demographics* items included gender (male, female), age (continuous in years), education (none, primary, secondary, tertiary; dichotomised into (0) primary or less (1) secondary or greater based on distribution), monthly household expenditures measured on a 6 point scale ranging from less than 10,000 Shillings (∼3 USD) to more than 500,000 Shillings (∼15 USD); dichotomised into (0) 14 USD or less and (1) 15 USD or greater, and occupation (dichotomised into fishermen and all other).

*Internalized HIV stigma* was measured by calculating the mean of six items adapted from the Internalized HIV Stigma Scale (Kalichman et al., 2009); which includes items such as: ‘Being HIV positive makes me feel dirty’ with response options ranging from (0) strongly disagree to (4) strongly agree (Cronbach's α = 0.63).

*Anticipated HIV stigma* items originated from the Chronic Illness Anticipated Stigma Scale (CIASS) (Earnshaw, Quinn, Kalichman, & Park, 2013). We calculated the mean of seven items assessing participant's expectations that they will be stigmatized/discriminated against for their illness, such as: ‘Someone at work will discriminate against you’, with response options ranging from (0) very unlikely to (4) very likely (Cronbach's α = 0.88).

*Perceived general health status* was measured by a single item from the Short-Form Health Survey (Ware, Kosinski, & Keller, 1996): ‘During the past 4 weeks, how much of the time has your physical health or emotional problems intervened with your social activities (like visiting with friends, relatives, etc.)?’ with response options ranging from (0) none of the time to (5) all of the time.

*Food insecurity* was measured by one item asking, ‘In the last month, approximately how many days have you or any member of your household not had enough food to eat?’ adapted from the Food Security Survey Module: Short Form (USDA, 2012). We dichotomise this variable into ‘no food insecurity’ (0 days) to ‘yes, food insecurity’ (1 or more days).

*Mobility* was measured by a single item: ‘In the past 12 months, have you travelled and slept away from this community?’ (yes/no) (Kwena, Camlin, Shisanya, Mwanzo, & Bukusi, 2013).

*Structural barriers to care* were measured by the mean score of a 16-item Likert scale assessing tangible barriers to accessing care, adapted for the present study's context from the HIV Cost and Services Utilization Study barriers to care scale (RAND Corporation). The scale includes items like, ‘The clinic or clinic outreach is too far from where I work or live’, with response options ranging from (0) strongly disagree to (4) strongly agree (Cronbach's α = 0.71).

*HIV Fatalism* was measured using the mean score of 7 items adapted from Meyer-Weitz's Fatalism scale (Meyer-Weitz, 2005), and includes items like ‘It was just a matter of time before I got HIV.’ Response options on a five-point Likert scale range from ‘strongly disagree’ (0) to ‘strongly agree’ (4) (Cronbach's α = 0.54).

*Engagement in transactional sex* was measured using two items: ‘Have you exchanged sex for money in the last year’ and ‘Have you exchanged money for sex in the last year.’ A response of no to both items was coded as (0) ‘no, did not engage in transactional sex;’ answering yes to one or both items is coded as (1) ‘yes, engaged in transactional sex.’

*Unprotected sex at last sexual intercourse* was measured by an item asking ‘Did you use a condom the last time that you had sexual intercourse?’ (yes/no) (Choudhry, Ambresin, Nyakato, & Agardh, 2015).

### Analysis

Using SPSS version 23 (IBM Corp, 2011), we use descriptive statistics and frequencies to assess participant characteristics, as well as the level and types of fatalistic beliefs among our sample. We use generalised logistic regression analyses to test the bivariate associations between HIV fatalism and the other independent variables (i.e. demographics: sex, age, education, monthly household expenditure, occupation; psychosocial factors: internalised and anticipated HIV stigma; structural factors: food insecurity, mobility) with our outcome of interest, engagement in transactional sex in the last year. We excluded unprotected sex as an outcome because of the small number of people reporting any condom use at last sex (*n* = 14). These factors were then included in a multivariate model to test the association of HIV fatalism and transactional sex, controlling for known and theoretically grounded risk factors for HIV. Similarly, we use generalised linear regression analyses to test the bivariate associations between the above-mentioned demographic, psychosocial, and structural factors variables, with the addition of perceived poor health (psychosocial) and structural barriers to HIV care (structural) (hypothesised only as predictors of fatalism) and HIV fatalism, followed by a multivariate model with all variables included. In each analyses, we controlled for HIV testing type as a covariate, as our prior research indicated significant socio-demographic differences between home-based versus event-based clients (Bogart et al., 2017).

## Results

### Participant characteristics and descriptive statistics

A total of 91 participants (51 female, 40 male) were included in the present analysis. The majority of participants had primary-level or no education (84.60%), and approximately 43% reported their monthly household expenditures below 15 USD. More than half of participants reported experiencing food insecurity (62.60%) in the last 30 days, and 56.20% reported travelling away from the community for work. The following occupations were represented in our sample: fisherman (30.76%), businessman/woman (27.47%), fishing support services (e.g. fish cleaner) (7.69%), sex worker (4.39%), and other (19.78%), including mainly ‘peasants’, farmers, housewives, bar attendants; 9.89% were unemployed. Sixty-seven percent of participants reported being married or living with a partner, while 22% were not married and 11% reported being separated or divorced. Only 15.40% of the sample reported using a condom at last sex, and 27.50% of participants reported engaging in transactional sex in the last year. With possible scores ranging from 0–3 on the HIV fatalism scale, the average score in our sample was moderate (mean = 1.50, *SD* = 0.51). Frequencies of responses for each scale item are reported in Table 1.

**Table 1.** Sample characteristics and descriptive statistics, Uganda 2015, *N* = 91.

	n (%)	Mean (SD)
**Demographics**		
HIV testing type		
Home	60 (65.9%)	
Event	31 (34.06%)	
Sex		
Female	51 (56.00%)	
Male	40 (44.00%)	
Age		31.93 (8.06)
Education		* *
Secondary	14 (15.40%)	* *
Primary or less	77 (84.60%)	
Monthly household expenditure		
15 USD or greater	52 (57.10%)	
14 USD or less	39 (42.90%)	* *
Occupation		* *
Fisherman	28 (30.76%)	* *
Businessman/woman	25 (27.47%)	* *
Fishing support services	7 (7.69%)	* *
Sex worker	4 (4.39%)	* *
Other	18 (19.78%)	* *
Unemployed	9 (9.89%)	* *
Marital status		* *
Married and/or living together	61 (67.00%)	* *
Not married, not living with someone	20 (22.00%)	* *
Separated/divorced	10 (11.00%)	* *
**Psychosocial factors**		* *
Internalised HIV stigma (possible range: 0–4)		0.98 (0.81)
Anticipated HIV stigma (possible range: 0–4)		1.26 (0.95)
Perceived poor health (possible range: 0–4)		1.14 (1.50)
**Structural factors**		* *
Food insecurity in the last 30 days		* *
Yes	57 (62.60%)	* *
No	34 (37.40%)	* *
Mobility (travel away from community)		* *
Yes	51 (56.0%)	* *
No	40 (43.90%)	* *
Structural barriers to HIV care (possible range: 0–4)		1.15 (0.49)
**HIV risk behaviour**		
Condom use at last sex		* *
Yes	14 (15.40%)	* *
No	77 (84.60%)	* *
Engaged in transactional sex in the last year (gave money or received money for sex)		* *
Yes	25 (27.50%)	* *
No	66 (72.50%)	* *
**HIV fatalism** (possible range: 0–3)		1.50 (0.51)
Life is risky and getting HIV is just one of those things.	59 (64.84%)	* *
It is better not to think about HIV and enjoy your life.	45 (49.45%)	* *
I do not care that I have HIV because it is just another disease.	41 (45.05%)	* *
I am more worried about dying from drowning, hunger or something other than HIV.	43 (47.25%)	* *
It was just a matter of time before I got HIV.	35 (38.46%)	* *
I will only worry about having HIV when I start to get ill.	31 (34.07%)	* *
Before testing positive, I had given up trying to protect myself from HIV.	27 (29.67%)	* *

Notes: SD = Standard deviation; Mean score of HIV Fatalism scale is reported, and the percentage of participants reporting ‘agree’ or ‘strongly agree’ for each individual item.

### Bivariate and multivariate logistic regression models for associations between independent variables and engagement in transactional sex

Adjusting for our covariate (HIV test site) and known HIV risk factors (demographics: sex, age, education, monthly household expenditure, occupation; psychosocial factors: internalised HIV stigma, anticipated HIV stigma; structural factors: food insecurity, mobility) in the multivariate model, HIV fatalism was statistically significantly associated with engagement in transactional sex; for every one point increase on the HIV fatalism scale, participants were more than three times as likely to report engaging in transactional sex in the last year (AOR = 3.07, 95% CI = 1.02–9.23, *p* = 0.04). In the multivariate model, gender was marginally significant, with women less likely to report transactional sex than men (AOR = 0.21, 95% CI = 0.04–1.14, *p* = 0.07). In bivariate models, reporting food insecurity was also marginally associated with engaging in transactional sex (AOR = 2.88, 95% CI = 0.98–8.45, *p* = 0.06), as was HBHCT compared to event-based testing (2.60, 95% CI = 0.87–7.79, *p* = 0.09); however, these associations were not significant in adjusted models (Table 2).

**Table 2.** Results of bivariate generalised logistic regression model for correlates of engagement in transactional sex in the last year, adjusted for HIV test site and multivariate logistic regression model for correlates of engagement in transactional sex in the last year, Uganda 2015 (*N* = 91).

	Bivariate logistic regression			Multivariate logistic regression		
	AOR (95% CI)	x^2^	*p*	AOR (95% CI)	x^2^	*p*
HIV fatalism	**2.65 (1.01–6.92)**	**3.96**	**0.05**	**3.07 (1.02–9.23)**	**3.98**	**0.04**
**Demographic factors**						
Sex						
Female	0.67 (0.26–1.72)	0.69	0.41	**0.21 (0.04–1.14)**	**3.26**	**0.07**
Male (reference)						
Age	1.00 (0.94–1.06)	0.03	0.87	0.95 (0.88–1.03)	1.60	0.21
Education						
Secondary	4.08 (0.48–34.54)	1.66	0.20	0.65 (0.13–3.27)	0.28	0.60
Primary or less (reference)						
Monthly household expenditure						
15 USD or greater	1.26 (0.41–3.92)	0.16	0.69	0.71 (0.17–3.06)	0.21	0.65
14 USD or less (reference)						
Occupation						
Fishermen	0.82 (0.30–2.28)	0.14	0.70	0.29 (0.05–1.72)	1.86	0.17
Other (reference)						
**Psychosocial factors**						
Internalised HIV stigma	1.01 (0.57–1.77)	0.00	0.98	1.04 (0.48–2.24)	0.01	0.93
Anticipated HIV stigma	0.82 (0.49–1.39)	0.55	0.46	0.62 (0.31–1.28)	1.67	0.20
**Structural factors**						
Food insecurity						
Yes	**2.88 (0.98–8.45)**	**3.70**	**0.06**	2.57 (0.76–8.71)	2.31	0.13
No (reference)						
Mobility (travel away from community)						
Yes	0.78 (0.30–1.99)	0.28	0.60	0.49 (0.16–1.52)	1.51	0.22
No (reference)						
**Covariate**	** **	** **	** **	** **	** **	** **
HIV testing type	** **	** **	** **	** **	** **	** **
Home	**2.60 (0.87–7.79)**	**2.91**	**0.09**	3.16 (0.61–16.24)	1.89	0.17
Event (reference)	** **	** **	** **	** **	** **	** **

Notes: HIV test site refers to the type of testing event in which participants were recruited for participation (home-based HIV testing or community-based event testing); AOR = Adjusted odds ratio; x^2^ = Wald chi square; Bivariate models are adjusted for HIV testing site only, multivariate model are adjusted for variables included in the model; Bolded text is used to indicate statistically significant associations at the *p* < 0.10 level.

### Bivariate and multivariate generalized linear regression for associations between independent variables and HIV fatalism

The independent variables positively associated with HIV fatalism at *p* < 0.05 level in bivariate models included: monthly income, internalised stigma, perceived poor health, mobility, and perceived structural barriers to HIV care. In the multivariate model, only greater structural barriers to HIV care remained significantly associated with increased HIV fatalism (*β* = 0.26, Std. error = 0.12, *p* = 0.04). Monthly household expenditure was marginally associated with HIV fatalism in the multivariate model; those reporting 15 USD or greater reporting higher HIV fatalism scores than those reporting 14 USD or less (*β* = 0.24, Std. error = 0.14, *p* = 0.09). (Table 3)

**Table 3.** Results of bivariate generalised linear regression model for correlates of HIV fatalism, adjusted for HIV test site and multivariate generalised linear regression for correlates of HIV fatalism, Uganda 2015 (*N* = 91).

	Bivariate linear regression			Multivariate linear regression		
Model	β	Std. Error	*p*	Β	Std. Error	*p*
**Demographic factors**						
Sex						
Female	0.12	0.11	0.25	−0.01	0.17	0.96
Male (reference)						
Age	−0.001	0.01	0.93	0.00	0.01	0.58
Education						
Secondary	−0.01	0.15	0.94	−0.08	0.16	0.61
Primary or no schooling (reference)						
Monthly household expenditure						
15 USD or greater	**0.30**	**0.12**	**0.01**	**0.24**	**0.14**	**0.09**
14 USD or less (reference)						
Occupation	** **	** **	** **			
Fishermen	−0.18	0.12	0.14	−0.67	0.19	0.73
Other (reference)	** **	** **	** **			
**Psychosocial factors**	** **	** **	** **			
Internalised HIV stigma	**0.14**	**0.07**	**0.03**	0.08	0.08	0.32
Anticipated HIV stigma	0.05	0.05	0.37	−0.00	0.07	0.97
Perceived poor health	**0.11**	**0.04**	**0.008**	0.07	0.04	0.11
**Structural factors**						
Food insecurity (prior 30 days)						
Yes	0.17	0.11	0.13	0.10	0.12	0.37
No (reference)						
Mobility (travel away from community)						
Yes	**0.22**	**0.11**	**0.03**	0.19	0.11	0.10
No (reference)						
Structural barriers to HIV care	**0.35**	**0.10**	**0.001**	**0.26**	**0.12**	**0.04**
**Covariate**	** **	** **	** **			
HIV testing type	** **	** **	** **			
Home	0.14	0.11	0.21	−0.81	0.17	0.64
Event (reference)	** **	** **	** **	** **	** **	** **

Notes: HIV test site refers to the type of testing event in which participants were recruited for participation (home-based HIV testing or community-based event testing); *β* = Beta, Std. error = standard error; Bivariate models are adjusted for HIV testing site only, multivariate model are adjusted for all variables statistically significant at the *p* < 0.10 level in bivariate models; Bolded text is used to indicate statistically significant associations at the *p* < 0.10 level.

## Discussion

Among Ugandan fisherfolk living with HIV, we found overall moderate levels of HIV fatalism. Consistent with qualitative studies, high proportions of our sample agreed that it was just a matter of time before getting HIV, it is better to not think about HIV, and that dying from other risks (drowning, hunger) is more worrisome than dying from HIV (Lubega et al., 2015; Sileo et al., 2016). This study uniquely contributes to the literature by identifying correlates of HIV fatalism. In multivariate models, HIV fatalism was associated with structural barriers to HIV care. Though not significant in the final model, significant associations with greater monthly household expenditure, internalised HIV stigma, poor perceived health, and mobility in bivariate models points to other psychosocial and structural variables that may influence fatalism. To our knowledge, our study provides the first quantitative evidence that HIV fatalism is associated with increased engagement in transactional sex among Ugandan fisherfolk. This association remained significant even after controlling for known HIV risk factors, including socio-demographics, psychosocial, and structural factors.

This research builds on previous qualitative studies that identify HIV fatalism as an important contributing factor to HIV risk behaviour among fisherfolk (Lubega et al., 2015; Seeley et al., 2009; Sileo et al., 2016). While the existing quantitative literature on HIV fatalism and risk behaviour have not examined transactional sex specifically, prior studies with men who have sex with men in developed countries demonstrate that individuals with fatalistic beliefs are more likely to engage in unprotected sex (Bland et al., 2012; Yi, Sandfort, & Shidlo, 2010). In sub-Saharan Africa, unprotected sex, number of partners, and faithfulness were not associated with HIV fatalism in Mali (Hess & McKinney, 2007); though greater AIDS education and having disclosed one's status was associated with less HIV fatalism among Malians and Gabonese (Hess & Mbavu, 2010). More work is needed to identify possible mechanisms through which fatalism may increase sexual risk. Prior work has suggested that HIV fatalism may lead to increased risk behaviour through skewed perceptions of risk or risk denial (Lubega et al., 2015; Mojola, 2011), which has been documented among Ugandan fisherfolk (Lubega et al., 2015), and should be explored further in similar populations.

We found that perceived structural barriers to HIV care was significantly associated with higher HIV fatalism in the multivariate model, supporting the hypothesis that HIV fatalism can result from inadequate access to HIV prevention and treatment services in communities with high HIV burden (Meyer-Weitz, 2005). The Ugandan MOH has prioritised ‘treatment as prevention’ through test-and-treat initiatives (i.e. the scale up of antiretroviral treatment [ART] regardless of disease stage) with fisherfolk and other high risk groups (Uganda MOH, 2013). Nonetheless, access to ART in these communities is limited due to distance from the clinic, mobility and working on the lake, and other structural barriers such as quality of care and long wait time at the clinic (Bogart et al., 2016; Seeley & Allison, 2005). Prior research in Malawi provides evidence that increased access to ART is accompanied with reduced HIV fatalism (Conroy, Yeatman, & Dovel, 2013). Given our study's finding that structural barriers to HIV care were associated with greater HIV fatalism, more work is needed to assess whether increased accessibility to ART could reduce HIV fatalism.

Several variables were significant in bivariate models but not the multivariate model, potentially because of our relatively low sample size (and thus, low statistical power), combined with their associations with other variables in the model. For example, perceived poor health and internalised HIV stigma were significantly associated with HIV fatalism in bivariate models. Fisherfolk who perceive their health as poor, but also perceive significant barriers to accessing HIV care, might subscribe to learned hopelessness about acquiring HIV and managing their disease. High levels of internalised stigma among fisherfolk living with HIV may further compound this hopelessness. HIV stigma in sub-Saharan Africa is thought to stem from its association with sexual promiscuity and immoral behaviour (Mbonu, van den Borne, & De Vries, 2009; Treves-Kagan et al., 2016). However, fatalistic beliefs about people with HIV being ‘dead before dying’ is an additional source of HIV stigma (Niehaus, 2007). Future research should more explicitly explore the intersection of HIV stigma and fatalism among fishing populations to better understand the bidirectional relationship between these two constructs.

In the bivariate model, fisherfolk who reported travelling away from the community were more likely to report greater HIV fatalism. Mobility has been identified as a barrier to HIV care and treatment among fisherfolk (Bogart et al., 2016); therefore, the association between mobility and HIV fatalism may be explained by structural barriers to care among fisherfolk who are mobile. In our sample, mobility may also be an indicator of social isolation or reduced social support, or greater exposure to occupational hazards, which are also thought to contribute to the high levels of HIV fatalism observed in fishing communities (Meyer-Weitz, 2005).

This study has several limitations. Our sample size may have limited our study's power to fully assess associations between the constructs studied, as well as the generalizability of findings to the broader population of fisherfolk living with HIV in Uganda. We do not know what effect participants’ recent HIV diagnosis may have had on their perceptions of fatalism, since participants were surveyed immediately after screening positive for HIV. It is possible that their fatalistic beliefs were elevated at the time of the survey. Another limitation is the modest reliability of our HIV fatalism scale (Cronbach's alpha = 0.54); reliability scores above 0.70 are preferred (Cortina, 1993). Nonetheless, we still found HIV fatalism, as assessed with this scale, to be associated with factors hypothesised to be associated with fatalism, such as structural barriers to HIV care. Several scales exist to measure HIV fatalism (Hess & Mbavu, 2010; Meyer-Weitz, 2005), but these scales need to be validated with Ugandan fisherfolk specifically.

## Conclusions

HIV fatalism may be an important correlate of engagement in transactional sex among Ugandan fisherfolk living with HIV. Since transactional sex is a driver of HIV transmission in sub-Saharan African fishing communities (Béné & Merten, 2008; Kwena, Bukusi, Omondi, Ng’ayo, & Holmes, 2012; MacPherson et al., 2012), this finding has implications for HIV risk reduction interventions in this setting. Our findings highlight HIV fatalism as a product of broader social and contextual factors, particularly structural barriers to HIV care, suggesting the need for structural interventions in this setting.
